# Case report of a medication error by look-alike packaging: a classic surrogate marker of an unsafe system

**DOI:** 10.1186/s13037-014-0047-0

**Published:** 2015-03-13

**Authors:** Joerg Schnoor, Christina Rogalski, Roberto Frontini, Nils Engelmann, Christoph-Eckhardt Heyde

**Affiliations:** Department of Anesthesia and Intensive Care Medicine, University Hospital Leipzig, Liebigstraße 20, 04103 Leipzig, Germany; Office of Quality and Risk Management, University Hospital Leipzig, Liebigstraße 20, 04103 Leipzig, Germany; Department of Anesthesiology, King’s College Hospital Clinics Abu Dhabi LLC, Abu Dhabi, UAE; Shining Towers, Mubarak bin Mohammed St, Khalidiyah, PO Box 129923, Abu Dhabi, UAE; Pharmacy, University Hospital Leipzig, Liebigstraße 20, 04103 Leipzig, Germany; Department of Orthopedics, Traumatolgy and Plastic Surgery, University Hospital Leipzig, Liebigstraße 20, 04103 Leipzig, Germany

**Keywords:** Patient safety, Look alike-sound alike, LASA, Medication error, Costs, High work load

## Abstract

**Background:**

The acronym LASA (look-alike sound-alike) denotes the problem of confusing similar- looking and/or sounding drugs accidentally. The most common causes of medication error jeopardizing patient safety are LASA as well as high workload.

**Case presentation:**

A critical incident report of medication errors of opioids for postoperative analgesia by look-alike packaging highlights the LASA aspects in everyday scenarios. A change to a generic brand of medication saved costs of up to 16% per annum. Consequently, confusion of medication incidents occurred due to the similar appearance of the newly introduced generic opioid. Due to consecutive underdosing no life-threatening situation arose out of this LASA based medication error.

**Conclusions:**

Current recommendations for the prevention of LASA are quite extensive; still, in a system with a lump sum payment per case not all of these security measures may be feasible. This issue remains to be approached on an individual basis, taking into consideration local set ups as well as financial issues.

**Electronic supplementary material:**

The online version of this article (doi:10.1186/s13037-014-0047-0) contains supplementary material, which is available to authorized users.

## Background

In a health care system based on lump sum payment (Diagnosis-Related Group, DRG), competing suppliers of health care are forced to optimize cost efficiency to generate revenue. Simultaneously, patient safety has become an overall goal for all parties involved, and can limit cost efficiency and hence revenue substantially.

Patient safety is at risk due to medication errors, and roughly 30% are due to similar packaging and labeling, as well as illegible handwriting. The term LASA (“look-alike sound-alike”) delineates a confusion of medication due to the similar labeling and packaging of different drugs, or similar labeling and packaging of the same drug containing different strengths.

These errors occur along the line of prescribing, preparing, distributing and administering medication. Various factors contribute to the LASA incident, and accounts for 7-20% of all medication errors [1-5]:

- illegible handwriting
- oral and vague prescription (“half an ampule”)
- incomplete knowledge of name of medication and substance
- newly released drugs in ever shorter periods of time
- similar packaging and labeling
- dismissing barcode technology at point of care
- similar clinical use of drugs
- similar dosage and concentration
- diverging concentration on similarly looking packaging
- displaying concentration in percent instead of numerical units
- dismissing the use of capital letters (ie. PENTObarbital versus PHENObarbital)
- confusing and/or no separate stocking of high risk medication

A typical case of LASA has been published recently [6].

To demonstrate the reciprocity of patient safety and cost efficiency, this true report of medication errors highlighted by the use of a Critical Incident Reporting System (CIRS) is utilized. Savings on changing to a generic brand are calculated and measures to avoid medication errors are demonstrated, as published by most recent guidelines.

## Case presentation

**Figure 1:**
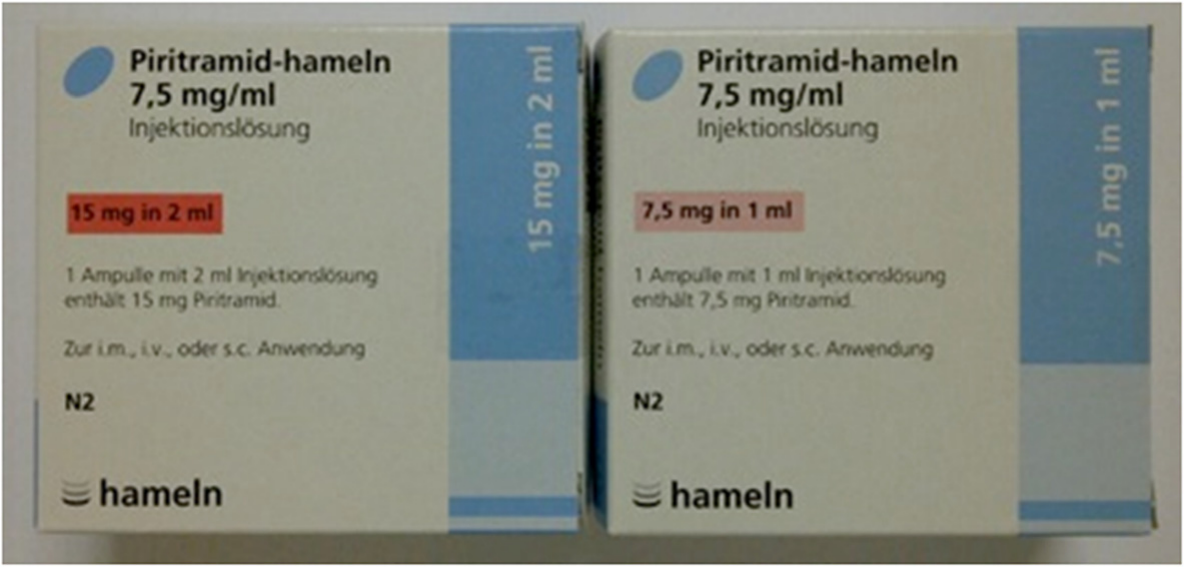
**Medication boxes of Piritramid-hameln (Hameln, Germany) with 2×5 vials ad 2 ml-vials (15 mg Piritramid) and 1 ml-vials (7,5 mg Piritramid), respectively.**

Upon restocking opioids, changing from Dipidolor™ (a trade name of Piritramid from the Janssen-Cilag GmbH, Germany) to the generic brand Piritramid (Hameln pharma, Germany), with Dipidolor being a commonly used opioid for post-operative pain in Germany, opioids were ordered as a 1 ml vial containing 7.5 mg/ml of Piritramid, instead of the usually ordered and used 2 ml vials containing 15 mg/2 ml of Piritramid, still from the same manufacturer (see the figure of medication boxes (Figure 1)). However, these stocks were falsely registered as 2 ml vials in the drug cupboard logbook on the ward. In some cases, prescriptions were made as “… administer half a vial of Piritramid…” by physicians. A CIRS was filed anonymously, however, at this point no conclusion could be drawn as how many patients had been involved and which patient had received what dosage. Assumingly “half an vial” of Piritramid has led to some patients receiving 3.75 mg of Piritramid instead of 7.5 mg.

As a learning system, the in-house CIRS is operating since 2010 on an electronic basis, voluntarily and anonymously. All cases are electronically published and archived. Two days after this case had been reported, both a review on anonymity and a request for proposals to the departments that may be involved were conducted. Two weeks later, the incoming suggestions on practicality were reviewed and, if possible, implemented.

### What recommendations have been published?

Apart from the regular requirements for staff, like holding a recognized degree in medicine or nursing, maximum attention to be paid when making difficult decisions on or distribution of any medication, or a sufficient number of staff to shoulder the workload, there are numerous recommendations [1]:

- interdisciplinary cooperation (pharmacist, physicians, auxiliary staff, commission on medication) regarding stocks of medication available
- non-standardized medication should be prepared by in-house pharmacy. Whenever feasible, ready to use/ ready to administer drugs should be given
- replacing similar sounding drugs by drugs with different brand name containing same substance maintain awareness for LASA issue. Keeping lists of such drugs, which can enhance awareness for the potential of medication error. Regular updates from the in-house CIRS should be mandatory
- if LASA prone medication is in use, these drugs should be highlighted by pharmacy or relabeled altogether (signal effect for potential danger of medication error)
- barcode scanning technology to be implemented at point of care
- all members of staff involved in medicating patients should receive recurring training for awareness of LASA
- stringent feedback of LASA issues to FDA and pharmaceutical industry via pharmacists from hospitals

A proven concept is the double verification principle or two-man rule. In Addition, there are various recommendations on logistics to reduce medication errors as well [7]:

- considering LASA when ordering stocks. Whenever feasible, preferring alternative suppliers
- ideally, only one strength/concentration of each substance should be available on wards. Diverging concentrations should be ordered according to individual cases only
- if, however, LASA medication needs to be stocked, these should carry warning labels, especially high risk medication with a narrow therapeutical margin, for example cardiovascular drugs, anesthetic drugs, cytostatins, high risk electrolyte carrying fluids, and the likes. Separate stocking of such drugs
- a change to a barcode driven medication process can reduce the risk of confusing drugs significantly [7,8]

### Previous changes in medication

Prior to this incident, a change was proposed from staff regarding usage of Piritramid 2 ml vials, as

- half of the substance (15 mg vials of 2 ml of Piritramid) was mostly discarded.

- cost efficiency could be achieved by switching to a generic product.

### Impact of change to a generic substance

**Figure 2:**
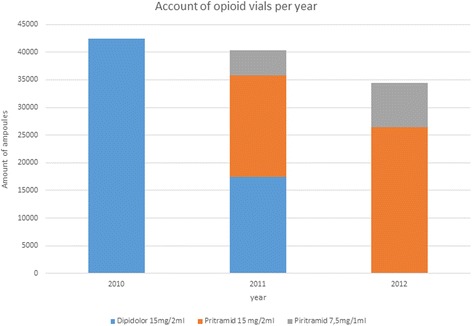
**Pharmacy dispensing of Dipidolor™- and Piritramid-vials in 2010–2012.**

The change to the generic Piritramid was implemented on June 6^th^ 2011. A pharmacy audit of 2010–2012 showed a reduction in prescribed Piritramid distribution (see the figure of pharmacy dispensing of Dipidolor™- and Piritramid-vials (Figure 2)). However, a multi-nodal pain bundle was implemented around that time as well, including an increase in regional anesthesia techniques intra-operatively, and could contribute to this downturn in post-operative opioid consumption of Piritramid considerably. Additionally, incomplete knowledge of newly introduced drugs and dosages could be adding to this phenomenon.

**Figure 3:**
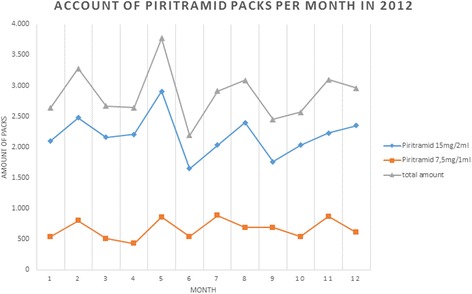
**Pharmacy dispensing of Piritramid-packs with 10 vials (7,5 mg in 1 ml; 15 mg in 2 ml) per month in 2012.**

Whether long-term effects based on an ageing population with a decreased need for opioids, or shorter overall stays in our hospital were attributing to this remains debatable, as pharmacy audit of 2012 showed a balanced consumption (see the figure of pharmacy dispensing of Piritramid-packs per month in 2012 (Figure 3)) again.

### Costs

A change in brands of stocked medication creates costs. Analysis of this process conducted by a University demonstrated costs of US$ 2.400 for a simple change to a generic substance.

A change to a generic substance with a different brand name amounted to US$ 5.200 on average. Introducing a different substance and name to replace a brand came with a price tag of around US$ 6.480 [9]. These costs are of course examples, whether these can be assigned to other changeovers remains to be seen.

Table 1 demonstrates a gross cost ratio of our given case (German prices). The observed pool of costs relating to Piritramid showed a reduction of 50% over the course of a three years’ time. During these three years of observation we found a decline in distribution of Piritramid of 19%. The change to a generic brand alone generated a cost reduction of 16% per year. This reduction in costs needs to be put in contrast to errors occurring in medicating patients with Piritramid 7.5 mg vials and Piritramid 15 mg vials.

**Table 1:** **Drug costs observed (List price 2012 in EURO; 1 Euro ~ 1,38 USD** [10]**)**

**Drugs (number of vials per package and costs)**	**costs 2010**	**costs 2011**	**costs 2012**
Dipidolor™ 15 mg/2 ml	n = 42.400	n = 17.500	n = 0
(5 vials 17,17€)	145.601,60 €	60.095,00 €	
Piritramid 15 mg/2 ml	n = 0	n = 18.300	n = 26.400
(10 vials 21,97€)		40.205,10 €	58000,80 €
Piritramid 7,5 mg/1 ml	n = 0	n = 4.500	n = 8.000
(10 vials 18,40€)		8280,00 €	14.720,00 €
Total costs (EURO)	145.601,60	108.580,10	72.720,80
**Total costs (USD)**	**200.930,21**	**149.840,54**	**100.354,70**

### In-house recommendations

As a rule, solutions must be tailored to local requirements while at the same time being financially reasonable. In the given case the 15 mg vials were taken out of stock completely. Alternatively, a different brand with a different label and packaging could have been chosen. If, however, different dosages are required, these could be spread over different wards/units alternatively, and staff awareness should be ensured, either computer based or by bulletins.

## Discussion

About 1/3 of all cases of confusing medication correlate with similar packaging and labeling of drugs, at the same time 50% of all cases of confusing medication is due to poor performance of qualified staff. Underlying reasons discussed are overly burdened staff and psychological aspects like confirmation bias [2,11-14]. Other problems like translational problems and mistakable labeling errors, which led to errors in the use of implants, were described and discussed in the recent literature [15].

A DRG based health care system payment coerces employees to exert more work in the same given time. This “rat race” leads to more pressure at the bedside, which might favor an increase in errors in medication. An analysis of 235 CIRS case reports within the Department of Anesthesiology showed that an overload of work is the second highest factor contributing to confusing medications as per LASA definition [16].

Sufficient recommendations do exist, targeting practical avoidance of medication errors/LASA. A financial compensation for this is not implemented in the German health care system as per DRG lump sum payment method, despite the fact that both politics as well as insurance companies should feel obliged to do so. Furthermore, pharmaceutical companies could contribute considerably to patient safety by abandoning a corporate design, reducing confusion of medication consecutively.

## Conclusion

Patient safety and cost efficiency do not necessarily have to exclude each other. Still targeting cost efficiency can easily endanger patient safety. Somehow both aspects can be seen as antipodes, where leveling can only be achieved for single cases, just before legal implications come into play.

Every hospital’s staff cannot handle the LASA issue individually, but politics as well as pharmaceutical companies can contribute significantly to patient safety regarding medication errors, especially by abandoning corporate design and allowing for a higher variety in packaging and design. Beside the LASA issue, understaffing contributes to confusion of medication and needs to be addressed locally, though determining the right amount of staff versus just not enough seems to be a thin line.

However, adding LASA to this gray area of understaffing and overload of work is an accident waiting to happen. Pressure on implementation of already existing measures to counter LASA experiences a new surge from indemnity insurers, forcing hospitals to invest in their staff and making sure that an ever-increasing workload is not detrimental to patient safety, finally leading to health care providers investing in patient safety.

## Abbreviations

DRG: Diagnosis-Related Groups
LASA: Look-alike-sound-alike
CIRS: Critical Incident Reporting System
